# Ratio effect slope can sometimes be an appropriate metric of the approximate number system sensitivity

**DOI:** 10.3758/s13414-019-01939-6

**Published:** 2020-01-13

**Authors:** Attila Krajcsi

**Affiliations:** Department of Cognitive Psychology, Institute of Psychology, ELTE Eötvös Loránd University, Budapest, Hungary

**Keywords:** Approximate number system, Number comparison, Ratio effect, Individual differences

## Abstract

The approximate number system (ANS) is believed to be an essential component of numerical understanding. The sensitivity of the ANS has been found to be correlating with various mathematical abilities. Recently, Chesney (2018, *Attention, Perception, & Psychophysics*, *80*[5], 1057–1063) demonstrated that if the ANS sensitivity is measured with the ratio effect slope, the slope may measure the sensitivity imprecisely. The present work extends her findings by demonstrating that mathematically the usability of the ratio effect slope depends on the Weber fraction range of the sample and the ratios of the numbers in the used test. Various indexes presented here can specify whether the use of the ratio effect slope as a replacement for the sigmoid fit is recommended or not. Detailed recommendations and a publicly available script help the researchers to plan or evaluate the use of the ratio effect slope as an ANS sensitivity index.

Mathematical thinking is essential not only in school, but also in everyday life, when making financial, economic, technical decisions. In past decades, it has been believed that one of the most important mental representations supporting mathematical thinking is the approximate number system (ANS; Dehaene, 1992). The ANS is a simple, analog, and noisy representation working according to Weber’s law (Moyer & Landauer, 1967). This system shows considerable individual differences, and these differences have been proposed to explain math grades in school (Halberda, Mazzocco, & Feigenson, 2008), mathematical competence measured with various tasks (Schneider et al., 2017), improvements of mathematical abilities (Park & Brannon, 2013), and numerical disabilities (Butterworth, 1999; Piazza et al., 2010). Therefore, it is essential to measure the sensitivity of the ANS in these types of works. However, recently it has been proposed that one of the indexes of the ANS sensitivity, the ratio effect slope, works incorrectly (Chesney, 2018). The present work extends this recent finding, offering additional viewpoints when one could or should not use the ratio effect slope to measure the ANS sensitivity.

## The measurement of the sensitivity of the ANS

**Fig. 1:**
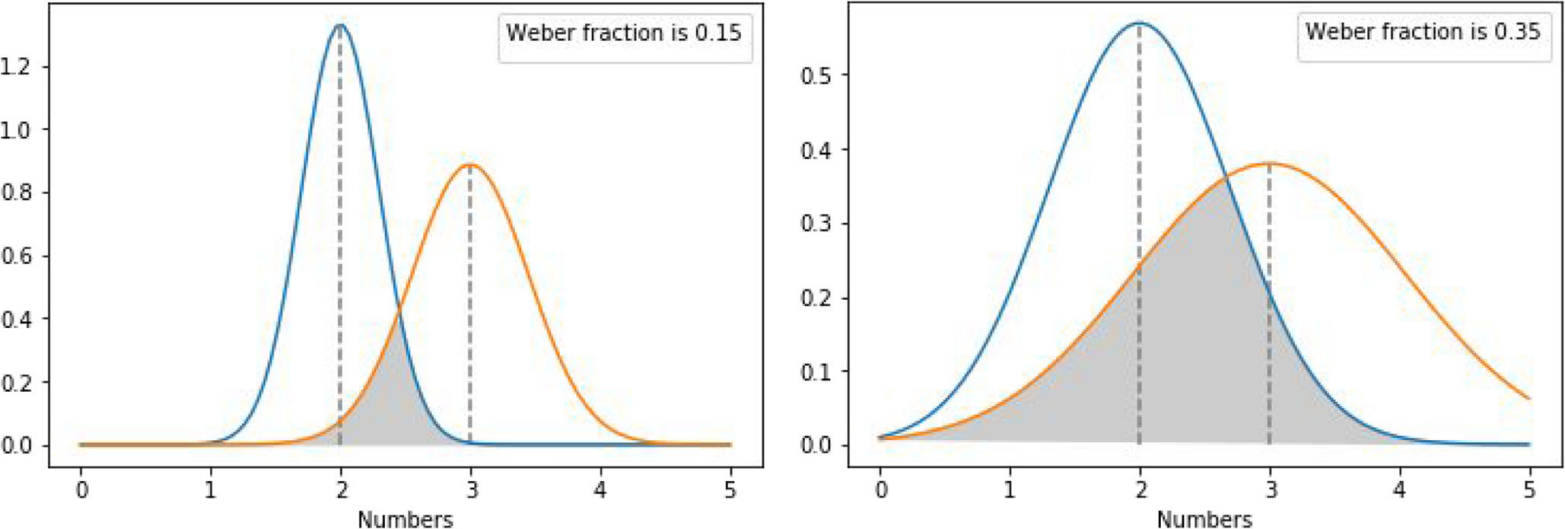
Overlapping noisy representations of values 2 and 3 with 0.15 (left) and 0.35 (right) Weber fractions

The ANS is a noisy representation that processes numerical values. The model follows former accounts of simple physical feature perception in psychophysics (Kingdom & Prins, 2010; Moyer & Landauer, 1967). In the ANS, numbers are stored as Gaussian distributions, and the size of the noise is described as the standard deviation (*SD*) of the normal distribution (see Fig. 1). In the ANS model, the standard deviation of the signal is proportional to the size of the number: Technically, in line with Weber’s law, the standard deviation is the product of the so-called Weber fraction and the value of the number to be represented. This Weber fraction is the sensitivity of the system: A more sensitive ANS has a smaller Weber fraction, and consequently smaller noise in the representation of the numbers. Weber fraction changes with age (e.g., Halberda & Feigenson, 2008; Piazza et al., 2010), and at any specific age it also shows individual differences (e.g., Halberda et al., 2008).

This simple model can explain many phenomena related to number processing. As a prominent phenomenon, the ANS can explain the ratio effect observed in a number comparison task. In a simple number comparison paradigm, the participant sees two numbers on the screen and has to choose the larger one. The performance of the typical participant is proportional to the ratio of the two numbers, termed the ratio effect: Number pairs with smaller ratio are harder to process, displaying higher error rate and higher reaction time (Dehaene, 2007). In the present work, ratio is defined as the larger number of the pair divided by the smaller number of the pair to be consistent with the work of Chesney (2018), whose work is extended here. (Note that many studies measure the distance and the size effects instead of the ratio effect; i.e., performance measured as a function of the difference or the sum of the numbers instead of the ratio of the numbers, but these effects are believed to measure the same ratio effect (Moyer & Landauer, 1967). In line with this consideration, in the present work, only the ratio effect is discussed, but the reasoning can be generalized to the distance and size effects.) According to the ANS account, the ratio effect is the consequence of the overlap of the two distributions (see Fig. 1): The smaller ratio of the to-be-compared numbers leads to a larger overlap, which in turn leads to worse performance. The model offers not only a qualitatively appropriate explanation for the ratio effect but also a relatively precise quantitative description of the number comparison performance (Dehaene, 2007; Moyer & Landauer, 1967).

As mentioned above, the model supposes that better sensitivity of the ANS means smaller Weber fraction, therefore smaller noise, which leads to more precise discrimination of the numerical stimuli, resulting in better numerical task performance. In a comparison task, for example, contrast the two panels in Fig. 1, where a Weber fraction of 0.15 (relatively high sensitivity) means a small overlap of the representations, leading to relatively good performance in the number comparison task, and where a Weber fraction of 0.35 means larger overlap with worse performance. This sensitivity is measured in many empirical works: Based on the performance, one can calculate the Weber fraction of a participant, then this Weber fraction can also be used to measure correlation with other mathematical tasks (Halberda & Feigenson, 2008; Halberda et al., 2008; Schneider et al., 2017).

There are two typical ways to measure the sensitivity (the Weber fraction) of the ANS in the literature. In the first method, error rates of number pairs with various ratios are measured, and a sigmoid function is fit to the data, measuring the spread of the sigmoid function as the Weber fraction (Dehaene, 2007; Kingdom & Prins, 2010). Note that it is not trivial how to measure the Weber fraction for reaction-time data, because the psychophysics models do not have a consensual prediction for reaction-time performance (Kingdom & Prins, 2010; Krajcsi, Lengyel, & Kojouharova, 2018), although see a possible solution in Palmer, Huk, and Shadlen (2005), Dehaene (2007) and Krajcsi et al. (2018).

In the second method for measuring the Weber fraction, the ratio effect is measured as the slope (i.e., weight) of a linear fit of the effect. In other words, comparison performance (error rate or reaction time) as a function of the ratio is calculated, and a linear fit is found for the calculated data, where the slope of the best linear fit is used as a measurement of the ANS sensitivity (Chesney, 2018; Schneider et al., 2017). (Note that the ANS model does not propose that the ratio effect should be described with a linear function. Instead, the model proposes to use a sigmoid function caused by the overlap of the distributions. Still, in the literature, the ratio effect [mostly measured as a distance effect] is frequently analyzed with a simpler linear fit, and the individual differences are often characterized by the slope of the linear fit.)^1^

Although there are several methodological works that empirically evaluate the psychometric properties of different metrics of the ANS sensitivity (Gilmore, Attridge, & Inglis, 2011; Holloway & Ansari, 2009; Inglis & Gilmore, 2014; Maloney, Risko, Preston, Ansari, & Fugelsang, 2010; Sasanguie, Defever, Van den Bussche, & Reynvoet, 2011), many of their conclusions might be questioned because of various methodological issues. For example, while measuring enumeration of nonsymbolic stimuli, many of those works use sets smaller than five (Gilmore et al., 2011; Holloway & Ansari, 2009; Maloney et al., 2010; Sasanguie et al., 2011), which small sets are subitized (i.e., they are enumerated relatively fast and with few errors rooted in a mechanism other than the ANS; Revkin, Piazza, Izard, Cohen, & Dehaene, 2008), and therefore, those indexes relying on these stimuli do not reflect ANS functioning validly. Another issue is that several of those works suppose that (in line with the original form of the ANS) both symbolic and nonsymbolic numbers are processed by the same system (Gilmore et al., 2011; Holloway & Ansari, 2009; Maloney et al., 2010), and the lack of the correlation between symbolic and nonsymbolic performance is interpreted as the invalidity of those indexes. However, according to some recent models, it might be possible that symbolic and nonsymbolic numbers are processed by different types of representations (see a summary in Krajcsi, Lengyel, & Kojouharova, 2016), and, therefore, the lack of the correlation of symbolic and nonsymbolic task indexes does not necessarily means that those indexes measure the ANS invalidly. Overall, though empirical works have tried to investigate the psychometric properties of the ANS metrics, additional works are required to find more valid evaluations of those indexes.

To summarize, the ANS is believed to be an essential representation behind several number processing phenomena, including the comparison ratio effect, and the sensitivity of the system is believed to be in large part the source of the differences in mathematical problem solving. The sensitivity of the ANS is typically measured in the number comparison task by either applying a sigmoid function fit that is common in psychophysics works, or by applying a linear fit on the ratio effect performance, while many psychometric aspects of those sensitivity indexes are still unknown.

## Problems when ANS sensitivity is measured as the ratio effect slope

**Fig. 2:**
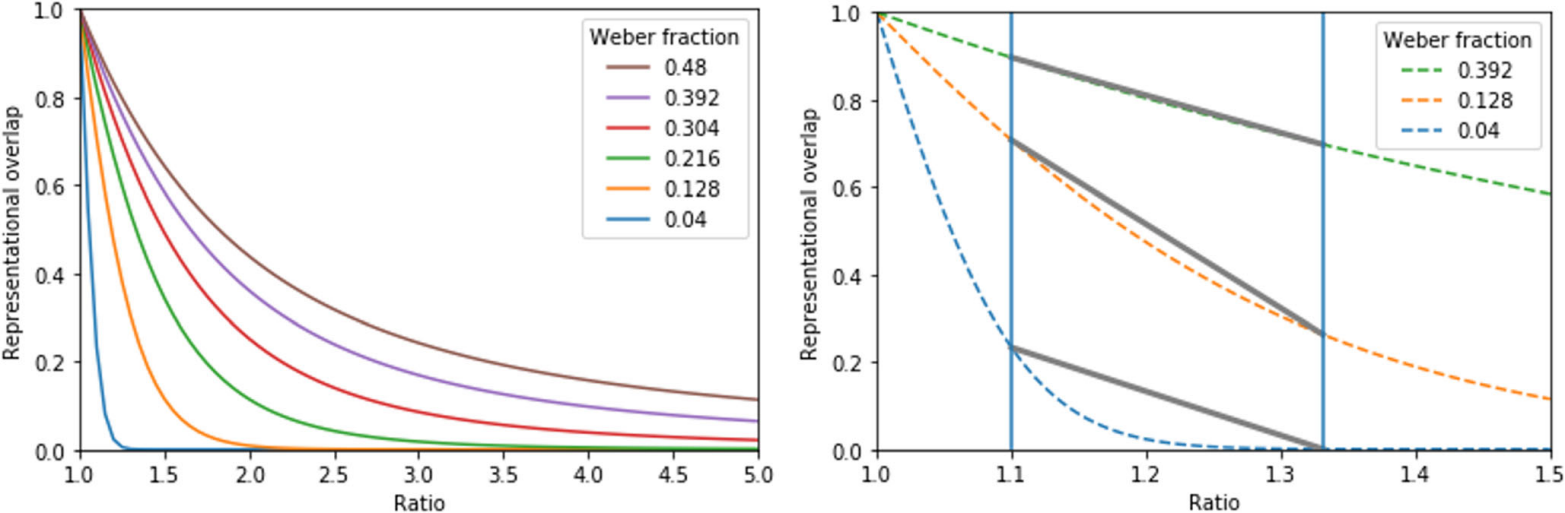
Overlap of two number representations in the ANS model as a function of the ratio and the Weber fraction. On the figure on the right, vertical lines denote chosen ratios in a hypothetical comparison task, and gray straight lines denote the ratio effect measured as linear performance change

**Fig. 3:**
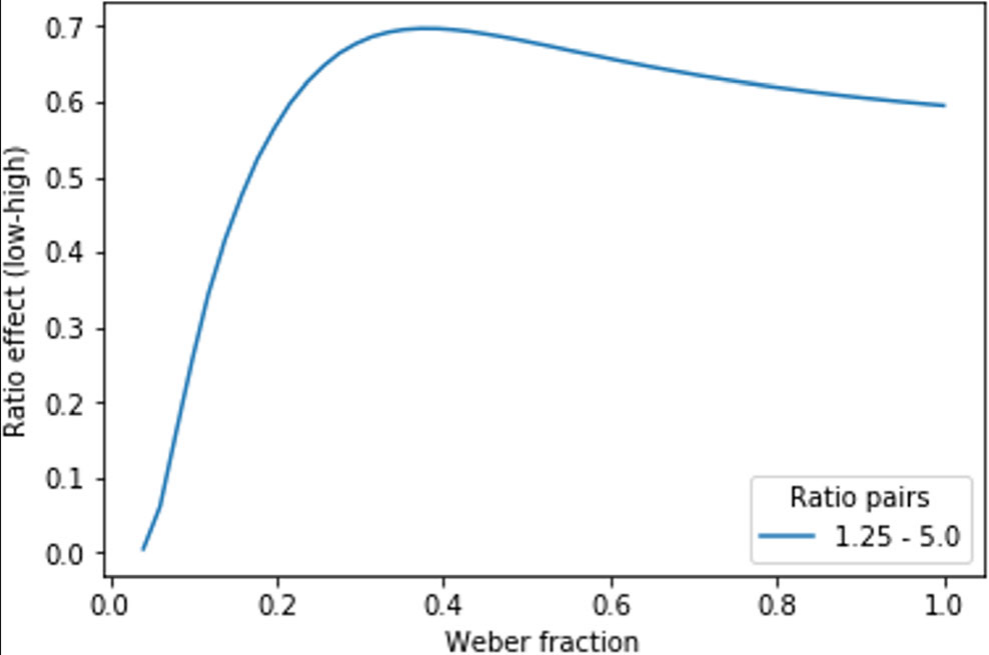
Ratio effect (calculated as a change in the representational overlap) as a function of the Weber fraction in comparison of pairs with 1.25 and 5 ratios

In an illuminating work, Dana Chesney (2018) demonstrated why the ratio effect slope may be misleading as a measure of the ANS sensitivity. Instead of measuring the psychometric properties of the ANS indexes empirically, such as the works described in the previous subsection, Chesney’s work investigated the mathematical relations of the ratio effect and the Weber fraction. According to the ANS model, in a comparison task, the overlap of the two noisy representations (and consequently the performance) depends on the ratio of the numbers to be compared and on the Weber fraction of the participant, and this overlap can be quantified. The left panel of Fig. 2 shows how the ANS model predicts the representational overlap as a function of the ratio of the numbers to be compared and the Weber fraction. (Figure 1 is made with the same algorithms as the simulations described below [see more details in the Method section], and Figs. 2–3 use similar parameters as the figures in Chesney, 2018, to illustrate the points of that work.)

The ratio effect can be calculated based on this function. In a simple ratio effect, the performance of two comparison tasks with two different ratios can be measured, and the performance change is the ratio effect. (Note that this might be considered as a special case of a linear fit when only two data points are used. In the present work, this index is referred to as the ratio effect slope, even if in this special case it is the difference of two data points. Still, the present reasoning can be generalized to cases where the ratio effect is measured with multiple ratios.) An example of the ratio effect can be seen in the right panel of Fig. 2, where vertical lines denote the used ratios in the comparison tasks, and gray straight lines denote performance change between the chosen ratios (i.e., the gray lines are the ratio effects). (Note that here, the representational overlap is considered instead of the measured behavioral indexes, i.e., the task difficulty is measured as representational overlap and not as error rate or reaction time. Still the relation of the model construct and the behavior indexes can be found, Dehaene, 2007; Halberda et al., 2008; Kingdom & Prins, 2010; Krajcsi et al., 2018, and the present reasoning can be generalized to those indexes as well.)

The problem with the linear fit of the ratio effect performance is that the slope does not always correlate with the Weber fraction in the same direction. For example, in the right panel of Fig. 2, for the Weber fraction 0.04 and 0.128 pair, as the Weber fraction increases, the ratio effect slope also increases. However, for the Weber fraction 0.128 and 0.392 pair, as the Weber fraction increases, the ratio effect slope gets lower (these parameters are similar to the parameters used in Chesney, 2018, Fig. 2d). To display the same problem in a different and a more general way, Fig. 3 demonstrates how the ratio effect changes as a function of the Weber fraction (the ratio effect is measured as representational overlap changes between two ratios, and the two ratios are 1.25 and 5, similar to the range in the second task in Chesney, 2018). The chart shows that for the small Weber fraction range, with increasing Weber fraction the ratio effect gets higher, whereas in the higher Weber fraction range, this relation is reversed.

Based on these properties, Chesney (2018) highlights that the ratio effect slope is an inappropriate measurement of the ANS sensitivity, because sometimes the ratio effect slope increases with higher Weber fraction, and in some other cases the ratio effect slope decreases with higher Weber fraction. Note that it is not trivial to recognize this nonmonotonic property: Researchers routinely used the ratio effect slopes to measure the ANS sensitivity even when this nonmonotonic relation is critical in the research (see examples summarized in Inglis & Gilmore, 2014; Schneider et al., 2017) or the relation is incorrectly considered to be monotonic even when the properties of those indexes are investigated (Inglis & Gilmore, 2014).

## Additional factors influencing the relation of the ratio effect slope and the Weber fraction

While the analysis of Chesney (2018) illustrates an essential property of the ratio effect slope, the main conclusion of that work might be too strong. Although in some cases the ratio effect slope will not reflect the ANS sensitivity linearly or even in a monotonic way, there could be scenarios where the ratio effect slope is closely linearly related to the Weber fraction. These details are critical when one has to decide which results of former works should be ignored and which results could be kept (such as in the meta-analysis of Schneider et al., 2017), or when one plans a new study to measure the ANS sensitivity with the ratio effect slope.

### Estimated range of Weber fractions in the measured population

First, there could be Weber fraction ranges in some groups where the relation of the ratio effect slope and the ANS sensitivity is close to linear, or at least monotonic. Former studies suggest that the mean of the Weber fraction can be estimated to be somewhere between 0.12 and 0.22 in adults: 0.22 and 0.2 (Study 1 and 2 in Chesney, Bjalkebring, & Peters, 2015), 0.108 (Halberda & Feigenson, 2008), 0.15 (Piazza et al., 2010), 0.12 and 0.17 (control and Munduruku participants in Pica, Lemer, Izard, & Dehaene, 2004), and the standard deviation of the sample was 0.06 and 0.07 (Study 1 and 2 in Chesney et al., 2015). (Note that there are only a few standard deviations of the samples available in the literature, because this descriptive is usually not relevant in those works, therefore it is not reported.) Based on these parameters, the Weber fractions of most adults could be included in the 0.0–0.4 range (the same range is observable in Fig. 5 in Piazza et al., 2010, where the whole distribution is displayed). Importantly, for the ratio pairs used in Fig. 3, in this 0.0–0.4 Weber fraction range, the relation of the ratio effect slope and the Weber fraction is close to linear, or at least monotonic. On the other hand, in the case of some other groups with other typical Weber fractions, the nonlinear relation of the ratio effect and the Weber fraction could be a serious issue. For example, 14-year-old adolescents were measured to have a mean Weber fraction of 0.279 with a 0.096 standard deviation (Halberda et al., 2008). Supposing that this population has normal distribution,^2^ 95.4% of the population could be between the range of 0.087 and 0.471 (i.e., mean ±2 *SD*). With this extended range, a considerable part of the distribution would fall into the range where, based on the ratio effect slope, it is impossible to tell the Weber fraction (e.g., on Fig. 3, for ratio effect slopes larger than 0.6, it is impossible to tell whether the Weber fraction is in the range of 0.3–0.4, or in the range of 0.4–1.0). Finally, for children, the mean Weber fraction is even larger (e.g., 0.525 for 3-year-olds, 0.383 for 4-year-olds; Halberda & Feigenson, 2008; 0.34 for 5-year-olds with an approximate range of 0.1–0.5; Piazza et al., 2010). When these ranges group around the peak of the function in Fig. 3, the ratio effect slope cannot be used. However, when the mean is large enough with relatively small range, thus, when most of the population is beyond the peak, the ratio effect is again a straightforward index to measure the ANS sensitivity, although the relation of the ratio effect slope and the Weber fraction is reversed.

To summarize, although in some populations the use of the ratio effect slope is not recommended, in other populations the ratio effect slope might be used as a proper approximation of the Weber fraction. If in the expected range of the Weber fraction and the ratio effect slope show a monotonic relation, then the ratio effect slope can be used, otherwise the use of the ratio effect slope is not recommended.

### Used ratios

**Fig. 4:**
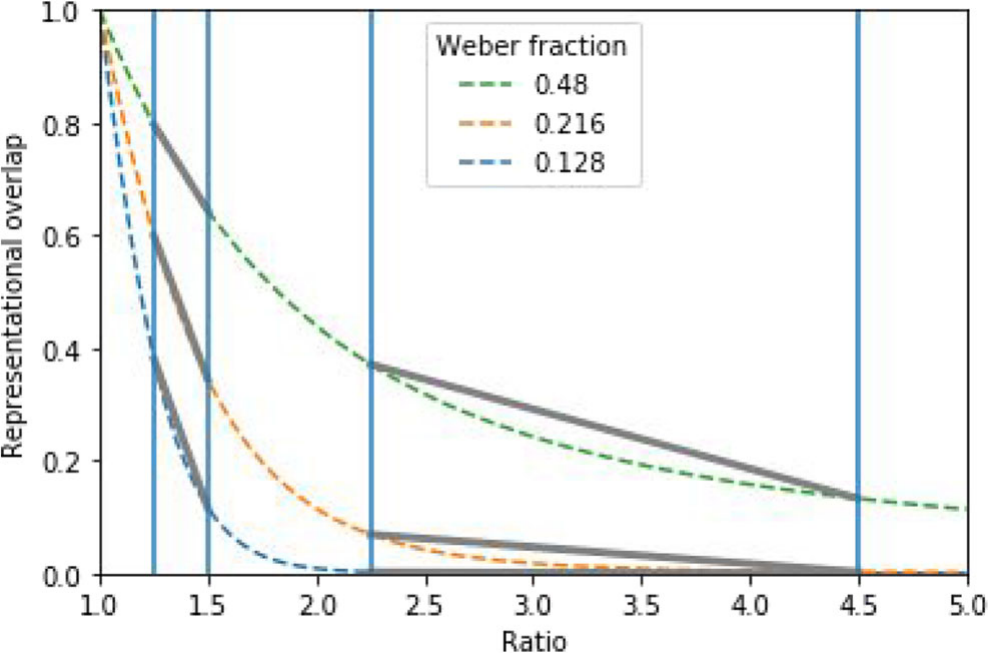
Overlap of two number representations in the ANS model as a function of the ratio and Weber fraction. Vertical lines denote chosen ratios, and gray straight lines denote the ratio effect measured as linear performance change

Another factor that might influence the usability of the ratio effect slope comes from the ratios of the number pairs in the comparison task. Looking at the relation of the representational overlap and the ratios (e.g., see Fig. 2), one might observe that those functions show an L shape: a relatively large drop of overlap as the ratio increases, then a smaller decrease for additional ratio increase. If two Weber fraction functions are on the same side of the corner of the L shape, then the correlation of the Weber fraction and the ratio effect slope is more straightforward (see Fig. 4): For the left part of the L-shaped functions, the ratio effect (e.g., between 1.25 and 1.5; the two vertical lines on the left) is increasing as the Weber fraction decreases. On the other hand, for the right part of the function, the ratio effect (e.g., between 1.75 and 4; the two vertical lines on the right) is decreasing as the Weber fraction decreases. Thus, in those examples, the relation of the Weber fractions and the ratio effect slopes are more straightforward. However, in some other ratio ranges, the relation of the ratio effect slope and the ANS sensitivity can be more complex, as demonstrated, for example, in Fig. 2. The relation of the two variables is more complex in this latter example because in that specific ratio range, depending on the Weber fraction, the representational overlap-ratio function could be either in the left or in the right or in both parts of the L-shaped function.

To summarize, depending on the ratios of the numbers and the Weber fraction range, the direction of the relation of the representational overlap and the Weber fraction could be either positive, negative, or mixed. This also means that while in some cases the ratio effect slope is not recommended to use, in some other cases it can be. Combining the issues of the expected Weber fraction range and the number ratio range, it is also critical that the two factors interact: The ratio and the Weber fraction ranges where it might be appropriate to use the ratio effect slope as an ANS sensitivity measurement depend on both factors at the same time.

### The effect of the distorted ratio effect slope on correlational studies

Depending on the Weber fraction range and the number ratios, the ratio effect slopes differ not only in whether the direction of the relation of them with the Weber fractions is positive, negative, or mixed but also in how much ratio effect slope range is involved. For example, in Fig. 4, for the 1.25–1.5 ratios the slopes include a larger range than for the 2.25–4.5 ratios. Thus, the expected ratio effect slope variability depends on the ratio used in the comparison task. As another factor influencing the expected variability, in Fig. 3, in the 0.0–0.3 Weber fraction range, the ratio effect includes a large ratio effect slope range between 0 and 0.7, while in the 0.4–1.0 Weber fraction range, the ratio effect slope includes only a small range between 0.6 and 0.7. Thus, the expected variability of the ratio effect slopes depends on the expected Weber-fraction range as well. Overall, the expected ratio effect slope variability depends both on the ratios of the numbers and the Weber fractions. This variability is relevant when running correlational studies, because with a supposedly constant noise smaller variability leads to a worse signal-to-noise ratio and worse reliability of the index.

The observed specific shape of the function of the ratio effect slope and the Weber fraction also influences correlational studies. The functions similar to the one seen on Fig. 3 clearly specify the relation of the Weber fraction and the ratio effect. The nonlinear relation obviously influences correlational studies when ANS sensitivity is measured with the ratio effect slope and this index is correlated with some other index, because the Pearson correlation coefficient measures the standardized goodness of fit of a linear regression, and deviation from linearity diminishes the observed correlation. One might investigate how strongly this nonlinear relationship of the ratio effect slope and the Weber fraction impoverishes the correlation of the Weber fraction and another property. The change of the correlation is not trivial because (a) in samples with normal distribution most cases are close to the mean, and relatively few cases are from the tails, and (b) cases from the tails may have a larger effect on the regression slope.

To summarize, the distorted measure of the ANS sensitivity with the ratio effect slope index has at least two ways to influence correlational studies. First, with the expected variability of the slopes it influences the reliability of the index. Second, the deviation from the linear relation between the ratio effect slope and the Weber fraction, the measured correlation of the ratio effect slope and another property could be lower than the correlation of the Weber fraction and the other property. To quantify the size of these effects, one should consider both the number ratios and the Weber fractions.

## Aims of the present study

The aim of the present study is to extend the analysis of Chesney (2018), and instead of suggesting mainly to avoid the ratio effect slope, it is investigated how (1) the expected Weber fraction range and (2) the used number ratio range influence whether the ratio effect slope is recommended to use or not by measuring (a) the expected ratio effect slope, (b) the expected range of ratio effect slopes in a group, and (c) the expected decay of the correlation coefficient in a correlational study. Beyond those main aims, (3) in the present simulation a more precise method is used to calculate the representational overlaps than was used by Chesney (2018; see the Method section for more details), and (4) a Python script is provided to offer a tool with which anyone can calculate the validity of the planned or already used ratio effect slope indexes. Finally, (5) a refined recommendation is given about when and how to use the ratio effect slope to measure the ANS sensitivity.

## Method

A computer simulation is performed in Python language, in a Jupyter Notebook environment. The notebook is publicly available at https://osf.io/69qnk/.

### Relation of the Weber fraction and the ratio effect slope as a function of ratio pairs

In a comparison task simulation, overlaps of two normal distributions were calculated. For a ratio effect slope, two overlaps for two ratios were calculated, and the ratio effect was calculated as the difference of the two overlaps. The ratio effect differences were calculated for various Weber fractions and various ratio pairs.

#### Representational overlaps

The representational overlap for a given ratio and a given Weber fraction was calculated as the overlapping parts of two normal distributions, where the means of the distributions were 1 and 1 × ratio (i.e., the to-be-compared numbers), and the standard deviations of the distributions were 1 × Weber fraction and 1 × ratio × Weber fraction (i.e., the product of the mean and the Weber fraction), respectively. Overlaps of the noisy representations were calculated as the integrals of the minimum of the two normal distributions. Note that this will offer a more precise result than the approximation used by Chesney (2018; see the Appendix of that work for the method used there).

#### Weber fractions

In the simulation, Weber fraction between 0.04 and 1.0 were used, similar to the range in Chesney (2018). This range includes Weber fractions from typical adults to small children and people living with dyscalculia (Piazza et al., 2010; see also the discussion of typical Weber fractions in the introduction above).

#### Number ratios

Unlike in Chesney (2018), more diverse pairs of ratios have been used. In the present simulation, some of the ratio pairs defined a range where the representational overlap-number ratio function (such as the functions in Fig. 2) includes the steep, left side of the L-shaped function for a wide range of Weber fractions. Some other ratio pairs defined a range where the overlap-ratio function includes the flat, right side of the L-shaped functions. Finally, some ratio pairs used mixed overlap-ratio function sections, including both steep and flat parts of the function. As another consideration, not only ratio pairs that were used in former studies were investigated, because there could be other smaller or extreme ranges of ratios where the representational overlaps still show visible changes, but are not used in studies where the ratio range was not optimized for the present viewpoint. For example, beyond the 2.0 ratio (see Fig. 4) there are still considerable changes in the overlaps, and the Weber fraction is related mostly monotonically to the ratio effect slope, but usually studies also include the stimuli with smaller than 2.0 ratio (i.e., it is not typical that for one-digit numbers a study would omit pairs with smaller than 2.0 ratio, such as 3–2, 4–3, 5–3, 6–4, 6–5, 7–4, 7–5, 7–6). Based on these considerations, the 1.125–1.4, 1.125–2.0, 1.125–4.0, 1.5–5.0, 2.0–5.0, 4.0–9.0 ratio pairs were used in the simulations.

### Expected range of the ratio effect slopes

To quantify the expected range of the ratio effect slope in a sample, the difference of the smallest and the largest ratio effect was calculated for various ratio pairs and for various expected Weber fraction ranges. For example, based on Fig. 3, in an experiment where ratios of 1.25 and 5.0 are used, one might reckon that for Weber fractions between 0.1 and 0.4 (typical adults) the ratio effect can be expected approximately between 0.2 and 0.7, leading to a 0.5 ratio effect range. Because the representational overlap can be between 0 and 1, and because the difference of the larger and smaller overlap in a ratio effect can also be between 0 and 1, this index can be considered as a standardized index of the ratio effect size variability.

In this simulation, the same ratio pairs were used as in the ratio effect slope–Weber fraction relation simulation described above. Weber fraction ranges of 0.03–0.31 (0.17 ± 2 × 0.07, i.e., mean ± 2 × *SD*) and 0.087–0.471 (0.279 ± 2 × 0.096) were chosen in the simulation, as a possible range for population of adults and adolescents (Chesney et al., 2015; Halberda & Feigenson, 2008; Halberda et al., 2008; Piazza et al., 2010; Pica et al., 2004).^3^

### Correlation simulations

**Fig. 5:**
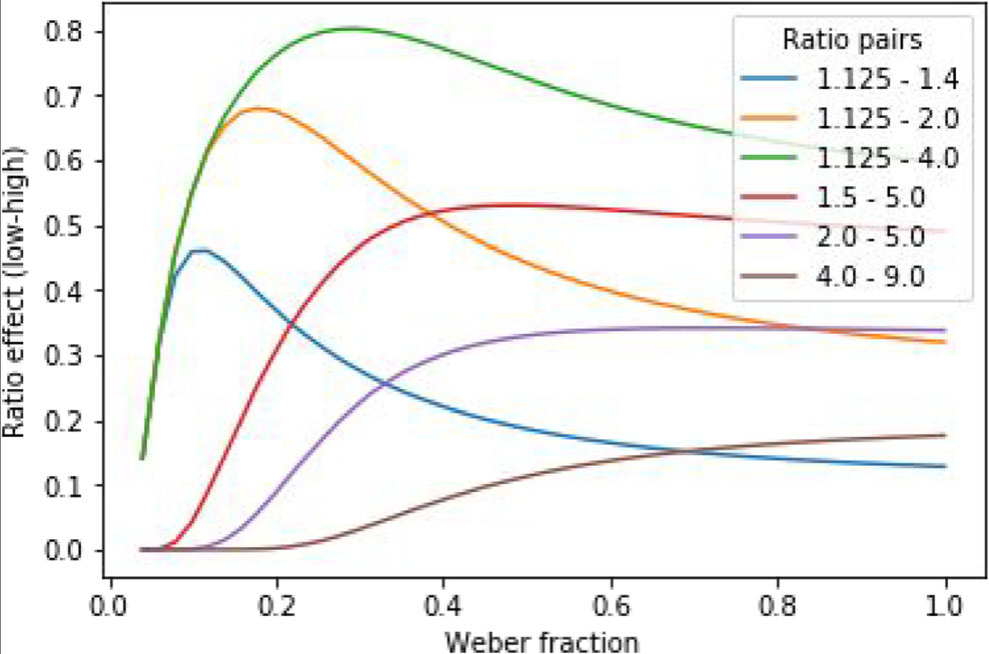
Ratio effect as a function of Weber fraction and ratio pairs

In the correlation simulation, it was measured what correlation could be observed if an index is perfectly correlating (i.e., *r* = 1) with the ANS sensitivity, but the ANS sensitivity is measured with the ratio effect slope. In a Monte Carlo simulation, samples of 50 participants were generated from a normal distribution.^4^ The correlation of the ratio effect slope and the Weber fraction was calculated (Weber fraction was considered as the index of a property that is perfectly correlating with the ANS sensitivity, which in turn is validly measured with the Weber fraction). (One can also consider this as sampling from a population showing a function displayed in Fig. 3 or Fig. 5, and the correlation of the two variables is calculated.) Sampling and calculation of the correlation was repeated 10,000 times, and the distribution of the correlations are displayed.

Samples with Weber fractions of a 0.17 mean and 0.07 standard deviation and of a 0.279 mean and 0.096 standard deviation were used to simulate a potentially typical adult and adolescent group, respectively. The ratio pairs of 1.125–2, 1.25–2, and 2–5 were used, to study various scenarios where the ratio pairs have potentially different predictions about the relation of the Weber fraction and the ratio effect slope (see the potential role of the number ratios on this relation described in the introduction).

## Results and discussion

### The effect of ratio pairs on the relation of the ratio effect slope and the Weber fraction

Figure 5 shows the ratio effect slope as a function of Weber fractions and ratio pairs. It is observable that the Weber fraction–ratio effect slope relation depends largely on the used ratio pairs. Ratio pairs of small values (such as the 1.125–1.4 ratios) lead to a relation mostly in the negative direction between the Weber fraction and the ratio effect slope, whereas ratio pairs of large values (such as the 4.0–9.0 ratios) lead to a relation mostly in the positive direction between the two ANS sensitivity indexes. Finally, ratio pairs of mixed (i.e., both small and large values; e.g., 1.125–4.0) values lead to a mixed sign direction relation between the two ANS sensitivity indexes in the wide 0.0–1.0 Weber fraction range.

The simulation clearly demonstrates that the relation of the Weber fraction and the ratio effect slope depends on the ratio pairs that are used in the comparison task and on the Weber fraction ranges one can expect in a sample, and the direction of the relation depends on whether the ratio pairs measure the representational overlap on the left or right part of the L-shape of the ratio and representational overlap function. Therefore, one should consider both the ratio pairs (or ratio range) and the expected Weber fraction range at the same time to tell whether the ratio effect slope index can be used to measure the ANS sensitivity or not, and, if the ratio effect slope can be used, to tell the direction of the ratio-effect-slope–Weber-fraction relation.

### Expected variability of the ratio effect slopes

The expected variability of the ratio effect slopes depending on the ratio pairs and the expected Weber fraction ranges are displayed in Table 1. Different ratio ranges show different expected variability, and the size of this variability or its maximum value depends on the Weber fraction range as well. Although as displayed in Fig. 5, the calculated range is simply the range of the ratio effect values (*y*-axis values) for a limited range of Weber fraction values (*x*-axis values), it is worth to quantify and report these values in studies, because the ratio effect is a nontrivial function of the number ratios and the Weber fractions.

**Table 1:**
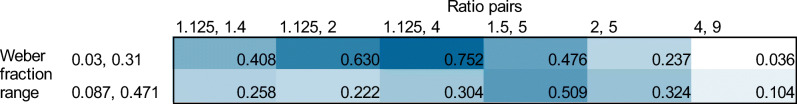
Expected range of ratio effects as a function of the ratio pairs and the expected Weber fraction range

*Note.* Darker cells depict larger ranges

In a study, the larger the expected variability of the ratio effect slope, the better it is, supposing that the relation of the ratio slope effect and the Weber fraction is monotonic or linear (see the next section, which quantifies this latter property in the context of correlational studies). The expected variability of the ratio effect size can be considered as an indirect index for the reliability of the ratio effect slope, because larger variability means larger signal-to-noise ratio if constant noise is supposed, therefore it also means better reliability of the ratio effect slope variable. Thus, the expected variability index may help to find a ratio that is optimal for a specific expected Weber fraction range of the sample. (Note that in groups with higher Weber fraction—e.g., children, participants living with dyscalculia—the noise is usually larger in the measured index. Therefore, the reliability of the indexes could be worse for them than for groups with a smaller Weber fraction. Still the expected variability within a specific Weber fraction range group can be compared across the ratio pairs and it can be considered as a component of the reliability of the ratio effect slope index.)

### The effect of a nonlinear relation of ratio effect slope and Weber fraction on correlational studies

**Fig. 6:**
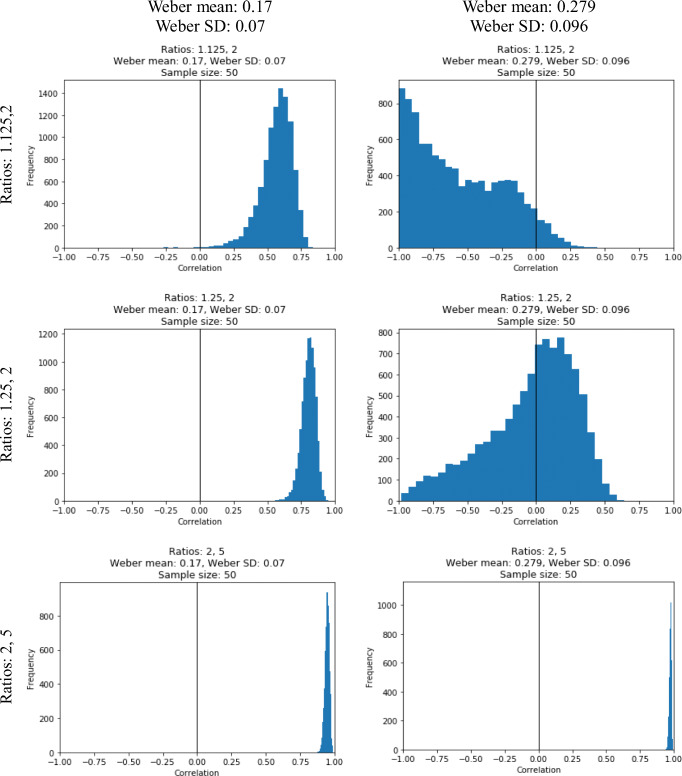
Distribution of the correlation coefficients (correlation of the ratio effect slope and a hypothetical property perfectly correlating with the Weber fraction) in simulated samples as a function of expected Weber fraction distribution (columns) and ratio pairs (rows)

Distributions of Pearson’s correlation coefficients as a function of ratio pairs (rows) and Weber fraction distribution (columns) are displayed on Fig. 6. It is clearly visible that the correlation heavily depends on the combination of the ratios and the Weber fraction distribution. For example, the 1.25–2.0 ratios with the typical adult Weber fraction distribution only slightly deteriorate the correlation, while the same ratios with the adolescent Weber fraction distribution ruin the correlation entirely. Although, as displayed on Fig. 5, the present correlation distributions simply depend on whether the function is linear in the range of the expected Weber fraction (*x*-axis values), it is still useful to estimate the expected effect of the deviation from linearity on the correlation.

Note that the narrow range of correlation coefficients does not necessarily means that the ratio effect slope will not influence the correlation compared with measuring the ANS sensitivity with the more direct sigmoid fit, because the expected variability of the ratio effect slope can be relatively small (see Table 1). In other words, the usability of the ratio effect slope in a correlational study depends both on the expected range of the slopes and on the correlation distributions: Usable ratio effect slopes have (a) high variability and (b) large absolute value with small variability of the correlational values. To put it another way, expressed in the features of the function displayed in Fig. 5, usable ratio effect slopes in a given expected Weber fraction range have (a) large differences in the ratio effects (*y*-axis values), and at the same time (b) there is a linear (or at least monotonic) relation between the ratio effect slope and Weber fraction.

## General discussion

The present work aimed to investigate the role of the number ratios on the relation of the Weber fraction and the ratio effect slope, and the effect of this relation on the correlations when ANS sensitivity is measured with the ratio effect slope. Former work has proposed that because of the nonlinear and even nonmonotonic relation of the ratio effect slope and the Weber fraction, the ratio effect slope is usually not an appropriate measure of the ANS sensitivity (Chesney, 2018). Here, it was found that in specific and well-defined cases, the ratio effect slope can be used as an appropriate measure of the ANS sensitivity, although one should specifically test the relation of the ratio effect slope and the Weber fraction for the used number ratio range. Similarly, it was found that the correlation is not necessarily flawed if the ANS sensitivity is measured with the ratio effect slope, although one should check the possible effect of the ratio effect slope with the specific number ratios and with the expected Weber fraction distribution.

Note that the present results do not mean that the studies using the ratio effect according to the present description necessarily measure the ANS sensitivity appropriately. Rather, the present work describes necessary but not sufficient conditions: Violating the required conditions leads to invalid measurement. However, even if meeting the present condition, additional properties of the measurement may ruin the metrics (e.g., small number of trials may lead to low reliability, or perceptual properties of the stimuli may influence the performance). The present work discusses only whether ratio effect slope is a usable index instead of the sigmoid fit method, and here, additional factors of the ANS measurement validity that are independent of the index calculation methods are not discussed in detail.

Because the present analysis investigated the mathematical properties of the ratio effect slope, empirical findings cannot overturn these properties. In line with this statement, the mathematical analysis proposed by Chesney (2018) or by the present work may explain some of the empirical findings investigating the psychometric properties of different ANS metrics. For example, mathematical analysis can explain why the ratio effect slope and the Weber fractions do not correlate strongly (Inglis & Gilmore, 2014), or why the ratio effect slope depends strongly on the measured ratios (Inglis & Gilmore, 2014).

Based on the present findings, refined suggestions are proposed for determining which index to use to measure the ANS sensitivity:

1. If it is possible, measure the Weber fraction with the sigmoid fit method instead of the ratio effect slope. Because a sigmoid function fit relies directly on the predictions of the psychophysics model, it can be considered as the most valid measurement of the ANS sensitivity, and therefore it is the recommended way of measurement. Note that several advanced and already accessible methods are available to recover the Weber fraction (Kingdom & Prins, 2010). While it is more straightforward to use a sigmoid fit method for error rates data (Dehaene, 2007; Halberda et al., 2008; Kingdom & Prins, 2010), it is also possible to find a workaround for reaction time data (e.g., using the diffusion model of decision-making; Dehaene, 2007; Krajcsi et al., 2018).
2. If the ratio effect slope is used, one can determine whether it gives an unbiased measure of the ANS sensitivity. The ratio effect slope is a relatively safe measurement for some ratio range and Weber fraction range combinations, whereas for other combinations the use of the ratio effect slope is not recommended. With the methods presented in this work, and potentially with the tool introduced below, one can determine whether the relation of the ratio effect slope and the Weber fraction is monotonic, and whether one can expect a sufficiently large variability in the sample.
  To ease the calculations that may be required when ratio effect slopes are used, the Python language Jupyter Notebook applied in the present work is made public (available at https://osf.io/69qnk/).^5^ To run similar simulations presented in this paper, but with modified parameters (other ratios, other Weber fractions, etc.), it is easy to find the appropriate parameters that should be modified in the Notebook, and to rerun the script.^6^
  With the method described here, one can decide if the ratio effect slope is appropriate to use. If the ratio effect slope is not appropriate to use, one can drop the study plan or the results. Alternatively, one can get a subset of the data (e.g., subset of ratios or subset of age range with more advantageous ratio effect slope properties) and reanalyze the data. This procedure can be used either for meta-analyses (such as in Schneider et al., 2017), where one should decide whether to include some results in the data set, or in study plans or reanalyses of former data to improve the properties of the ratio effect slopes. It is also recommended to include this information in the report of those studies.
  As mentioned above, the present considerations are only necessary, but are not sufficient conditions to use the ratio effect slope. Additional properties, independent of the index calculation method, might influence whether the ratio effect slope characterizes the ANS sensitivity correctly, such as the reliability of the indexes (Gilmore et al., 2011; Inglis & Gilmore, 2014; Maloney et al., 2010; Sasanguie et al., 2011), or perceptual, nonnumerical properties of the stimuli (DeWind, Adams, Platt, & Brannon, 2015; Gebuis & Reynvoet, 2012).

3. According to recent findings, symbolic numbers are not processed by the ANS, but by some other systems (e.g., see Lyons, Ansari, & Beilock, 2015). For example, Krajcsi et al. (2016) proposed that symbolic numbers are processed by the discrete semantic system (DSS), a network similar to the mental lexicon or conceptual networks. In the DSS model, size effect is a frequency effect, where smaller numbers are more frequent than larger numbers (Dehaene & Mehler, 1992), thus, smaller numbers are easier to process. Additionally, the distance effect is the consequence of the strength of associations between the digits and the smaller–larger categories (Krajcsi & Kojouharova, 2017). Importantly, unlike in the ANS model, in the DSS model, the comparison size and distance effects are not the consequences of the ratio effect, but are two independent effects rooted in different mechanisms. If one supposes that symbolic stimuli are not processed by the ANS, then the Weber fractions should not be used, but the indexes in line with the model should be used. For example, according to the DSS model, instead of the Weber fraction, the distance and size effect should be measured. (Note that unlike in the rest of this paper, in the context of DSS model, the distance and size effects are not considered as equivalents of the ratio effect as supposed in the ANS model, but they are indexes of two different mechanisms. Also, ratio-based performance change in symbolic number comparison is a meaningless and invalid parameter in the DSS model.)
  This third possibility also highlights that the present considerations about the ratio effect slope properties apply only if the ANS model is correct. If in any task or phenomena the ANS model is incorrect, then measuring the ANS is misleading, and obviously the ratio effect slope is also an invalid metric. In those cases, metrics that fit a more appropriate theoretical construct should be used. In line with those considerations, the present work only investigates what some necessary conditions are to test the ANS model validly, but the present work cannot tell if the ANS model is correct or not.

To conclude, in some specific and well-defined cases, the ratio effect slope can be used to measure the ANS sensitivity. Still, because of the nontrivial relation of the ratio effect slope and the Weber fraction, one must verify that the applied number ratios in a numerical task and the expected Weber fraction distribution of the participants ensure the validity of the ratio effect slope as an ANS sensitivity measurement.

## Footnotes

1 Note that as a third methods, the error rates can also be used (Inglis & Gilmore, 2014); however, this method largely depends on the parameters of the used paradigms.

2 But see some results demonstrating nonnormal distribution of Weber fractions in Inglis and Gilmore (2014).

3 Note that the Weber fraction distribution parameters used in the range of the slope simulation and in the correlational simulation do not intend to exactly represent adult or adolescent populations, but rather they represent reasonable examples of those groups.

4 Note that some measurements show that the distribution of the Weber fractions may deviate from normal distribution, showing some positive skewness (see Fig. 2 in Inglis & Gilmore, 2014; Fig. 5 in Piazza et al., 2010).

5 The use of the Notebook supposes that the user knows how to open, edit and run a Jupyter Notebook with a Python script. If the reader is not familiar with Jupyter Notebook or with Python, it is relatively easy to find various tutorials and descriptions explaining the basics of their use.

6 Note that the Notebook can be run either locally or on a cloud service.
